# Metabolic syndrome among patients with severe mental illness attending a tertiary hospital in northwestern, Tanzania: A cross sectional study

**DOI:** 10.1371/journal.pmen.0000573

**Published:** 2026-03-10

**Authors:** Didas Raymond Msele, Eligi Karol Kimosso, Florian Emanuel Ghaimo, Samuel Chacha, Noel Mrema Kilonzo, Matiko Mwita, Samuel Likindikoki

**Affiliations:** 1 Department of Psychiatry and Mental Health, Muhimbili University of Health and Allied Sciences, Dar es Salaam, Tanzania; 2 Department of Psychiatry and Mental Health, Catholic University of Health and Allied Sciences, Mwanza, Tanzania; 3 Department of Psychiatry and mental health, Bugando Medical Centre, Mwanza, Tanzania; 4 Bugando Medical Centre, Mwanza, Tanzania; 5 Department of Psychiatry and Mental health, KCMC University, Moshi, Tanzania; 6 Department of Epidemiology and Biostatistics, School of Public Health, Xi’an Jiaotong University Health Science Centre, Xi’an, Shaanxi, China; PLOS: Public Library of Science, UNITED KINGDOM OF GREAT BRITAIN AND NORTHERN IRELAND

## Abstract

Patients with severe mental illness exhibit a significantly higher prevalence of metabolic syndrome, with a risk approximately two times greater than that of the general population. This elevated risk may be attributed to factors such as the mental illness itself, the use of psychotropic medications, obesity, high-fat diets, low levels of physical activity, and smoking. The study aimed to determine the prevalence of metabolic syndrome and its associated factors among patients with severe mental illness attending Bugando Medical Centre [BMC] in Mwanza, Tanzania. This cross-sectional study included adults aged 18 years and above who attended the psychiatric clinic at BMC. Systematic random sampling was used. Data were collected using a structured questionnaire. Data analysis was performed using STATA version 17. Ethical approval was granted by the Institutional Review Board of MUHAS. In addition, permission to conduct the study was granted by the Director General of BMC, and written informed consent was obtained from all participating patients. A total of 305 patients participated in the study, with a mean age of 38.5 ± 14.2 years (range: 18–90 years). More than half of the participants [58.7%] were male. Metabolic syndrome (MetS) was identified in 33.1% of the participants. Increasing age was significantly associated with metabolic syndrome (MetS); participants aged ≥45 years had fivefold higher odds of having metabolic syndrome (MetS) compared with those aged 18–24 years [AOR 5.15, 95%CI: 1.55 – 17.16; P ≤ 0.008]. Three out of ten participants with severe mental illness were found to have metabolic syndrome, indicating a relatively high prevalence in this population. Increasing age was significantly associated with the precence of metabolic syndrome. Routine and frequent screening measures should be emphasized for the aging population with severe mental illness. A multi-disciplinary approach is essential to ensure comprehensive and holistic management.

## Introduction

The global burden of metabolic syndrome and its component is increasing. Metabolic syndrome has been shown to be associated with psychiatric illnesses [1–4]. The prevalence of metabolic disorders among patients with schizoaffective disorder, bipolar affective disorder, recurrent depression, and schizophrenia has been reported to range from 19–63%, 42.4%, 12–36%, and 8–56%, respectively [5–7]. In this population, factors beyond the mental illness itself may contribute to the development of metabolic syndrome including the use of antipsychotic medications, obesity, high-fat diets, physical inactivity, and smoking [8]. Overall, the prevalence of metabolic syndrome and its components among patients with Severe Mental Illness [SMI] is nearly twice that observed in the general population [9–11].

Metabolic syndrome [MetS] has gained increasing attention in psychiatric literature, as cardiovascular disease [CVD] is recognised as the leading cause of premature mortality among patients with severe mental illness [SMI] in developed countries [12,13]. Numerous studies have reported on the prevalence of metabolic syndrome and its associated risk factors among individuals with SMI worldwide [8,14]. In a meta analysis by Mitchell and colleagues, which included data from 27 countries collected between 2003 and 2011, the pooled prevalence of metabolic syndrome among patients with schizophrenia and related disorders was 32.5% [15]. Notably, no studies from Africa were included in this meta-analysis. Infact, approximately 0.001% of patients enrolled in schizophrenia clinical trials globally originated from Africa [15].

There is limited information on the prevalence of metabolic syndrome among patients with SMI in Africa. Mensah and colleagues reported that between 1990 and 2013, cardiovascular disease (CVD)–related deaths in sub-Saharan Africa (SSA) increased by 81%, with higher mortality observed among women than men [16]. These findings underscore the importance of generating region-specific data on the prevalence and risk factors of metabolic syndrome among populations in Africa. To the best of the authors’ knowledge, no studies have been conducted in Tanzania to determine the prevalence of metabolic syndrome among patients with SMI. Early identification and managemnet of metabolic syndrome in this population may help prevent cardiovascular and other associated complications.

This study is significant given the increasing prevalence of non-communicable diseases, particularly cardiovascular disease arising from metabolic syndrome. To our knowledge, this is the first study conducted in Tanzania among patients diagnosed with severe mental illness to determine the prevalence of metabolic syndrome. The study aims to establish the prevalence of metabolic syndrome and identify associated factors among patients with severe mental illness attending Bugando Medical Centre (BMC) in the Western Lake Zone of Mwanza. The findings will provide baseline data on the burden of metabolic syndrome in this population and highlight key risk factors associated with its development. Ultimately, these data may inform strategies to reduce cardiovascular, endocrinological, renal, hepatic, and hematological complications among patients with severe mental illness.

## Methods

### Study design and setting

This was a hospital-based cross-sectional study that employed a quantitative primary data collection approach. Data were collected from a sample of the target population between August and Novemeber 2024, and all selected participants were assessed.

This study was conducted at the psychiatric outpatient clinic within the Medical Outpatient Department of Bugando Medical Centre [BMC] in Nyamagana District, Mwanza, Tanzania. BMC is a tertiary, teaching, consultancy, and zonal referral hospital with an estimated 1000-bed capacity, serving the Lake Zone regions and a catchment population of approximately 13 million people. The hospital operates 24 hours for inpatient services and two days per week for outpatient clinics. Daily, around 40–80 patients are admitted to the inpatient department, while approximately 30–90 patients are attended at the Psychiatric Outpatient clinic, which is staffed by psychiatric nurses, medical doctors, and psychiatrists.

BMC was selected for its large catchment area covering eight regions, ensuring a diverse patient population and a representative sample of individuals with severe mental illness in the Lake Zone. The eligibility criteria included: 1) age ≥ 18 years, 2) a diagnosis of severe mental illness, and 3) attendance at the outpatient clinic during the data collection period, with provision of informed written consent. Participants were excluded if they had eaten within eight hours prior to the clinic visit, were experiencing acute symptoms, or had severe mental illness secondary to general medical conditions or substance use. Participants provided both verbal and written informed consent. Ethical approval was obtained from the Muhimbili University of Health Allied Science[MUHAS-REC-05-2024-2285] and permission to interview patients was granted by the Director General of BMC. Participants found with metabolic syndrome were reffered to medical outpatient clinic for further evaluation and care.

### Sample size estimation and sampling procedure

The minimum sample size of 305 was estimated using Cochran’s [1977] formula, based on a previous study conducted in South Africa, which reported a prevalence of metabolic syndrome (MetS) of 23.2% [16] and adjusting for a 10% non-response rate. Systematic random sampling was used to select patients for enrollment during clinic days (Tuesday and Thursdays). The clinic typically attends more than 80 patients per day, and the researcher aimed to interview 20 patients per clinic day. Therefore, the sampling fraction was calculated as the total number of patients attending the clinic divided by the number of patients to be interview [80/20]. The first patient card was selected randomly from the daily clinic attendees using a simple random technique.

### Data collection instruments and procedures

A structured questionnaire, developed in both English and Swahili, was used to collect demographic and clinical information. Demographic data included age, sex, marital status, educational level, employment status, residence, distance from the hospital, and health insurance status. clinical information collected included duration of illness, number of hospital admissions, type of psychotropic medication currently used, body mass index (BMI), Blood pressure and fasting blood glucose levels.

Metabolic syndrome (MetS) was defined according to the National Cholesterol Education Program Adult Treatment Panel III (NCEP ATP III) criteria as the presence of three or more of the following: (1) abdominal obesity—waist circumference (WC) >102 cm in men and >88 cm in women; (2) hypertriglyceridemia—triglycerides ≥150 mg/dL (1.695 mmol/L); (3) low high-density lipoprotein (HDL) cholesterol; (4) elevated blood pressure; and (5) elevated fasting glucose. Participants meeting these criteria were categorized as having MetS.

*Anthropometric measurements were obtained by trained medical doctors and nurses using standardized methods. Body weight was measured to the nearest 0.1 kg using a digital scale (Seca, Germany), and height to the nearest 0.1 cm using a stadiometer fixed to the clinic wall (Seca, Germany). Waist and hip circumferences were measured with a non-stretchable tape to the nearest millimeter. All measurements were conducted in triplicate, and the average was used for analysis. Waist circumference was used to assess abdominal obesity per NCEP ATP III criteria (≥88 cm for women, ≥ 102 cm for men), and body mass index (BMI) was calculated as weight in kilograms divided by height in meters squared (kg/m²).*

*Blood pressure was measured following a standardized protocol: participants rested for at least five minutes, and three serial measurements were taken one minute apart using a digital sphygmomanometer (CH-432B, Citizen Systems, Japan) with participants in a seated position. Average systolic ≥130 mmHg or diastolic ≥85 mmHg was considered elevated for MetS criteria.*

*Venous blood samples (10 mL) were collected and analyzed at the BMC laboratory. Triglycerides and HDL cholesterol were measured using a chemistry analyzer (ERBA XL, S.R.O Mannheim, Germany). Triglycerides >1.7 mmol/L and HDL < 1.03 mmol/L in men or <1.29 mmol/L in women were considered abnormal. Fasting blood glucose (FBG) was measured using a Hemocue 201 RT (Hemocue AB, Sweden); FBG 6.1–7.0 mmol/L was classified as impaired, and FBG > 7.0 mmol/L as diabetes. Participants with significant derangements in blood glucose or blood pressure were planned to be referred to the emergency department for stabilization, though no such cases occurred during data collection*

### Data processing and analyses

Data from the paper questionnaires were entered into the Microsoft Excel, and the original copies were secured stored in a locked cabinet. Data analysis was perfomed using STATA for Windows, version 17. Following data cleaning, descriptive analyses were conducted to summerise the demographic and biopsychosocial characteristics of the sample. Participants meeting three or more NCEP ATP 111 criteria were classified as having metabolic syndrome. Univariable analyses were perfomed, and results were presented in tables using means, frequencies, and ranges for each variable.

Bivariable logistic regression was performed to assess the associations between potential factors and the outcome of interest [metabolic syndrome]. Multivariable logistic regression was subsequently conducted to control for confounding variables. All factors were included in the multivariable model regardless of the p-values, as previous studies have demonstrated their potentail influence on the outcome; this approach ensured consistency with existing literature [17]. Variables with a p-value < 0.05 were considered statistically significant.

## Results

### The demographic and biopsychosocial characteristics of the study participants

A total of 305 participants were recruited in this study, with a mean age of 38.5 ± 14.2 years (range: 18–90 years). More than half of the participants were male [176; 58.7%]. Nearly half [152; 49.8%] were aged 18–35 years. Four out of ten participants [134; 43.9%] were single and more than one-third of the patients [121; 39.7%] had a primary level of education. The majority of the participants [231; 75.7%] were unemployed.

**Fig 1:**
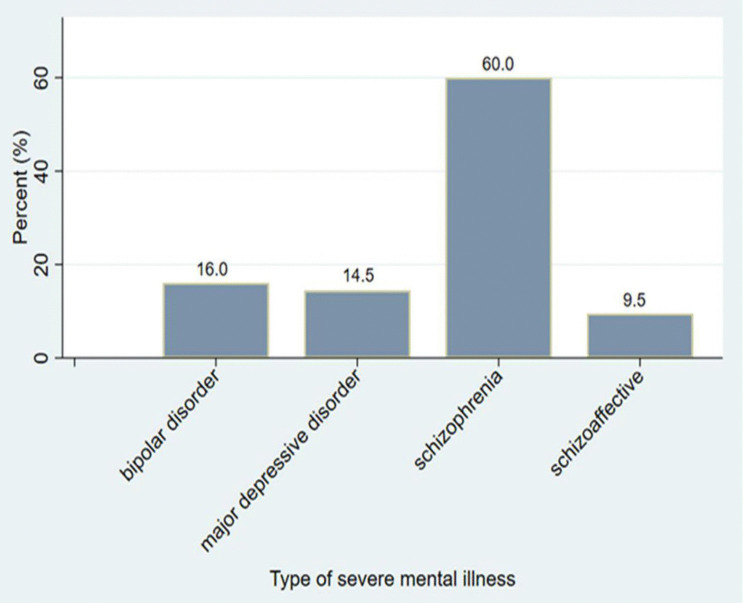
Type of severe mental illness.

Two-third of participants [201; 65.9%] lived ≥11km from BMC. Schizophrenia was the most common diagnosis, affecting six out of ten participants [183; 60.0%], while fewer participants had bipolar disorder [49; 16.1%], major depressive disorder [44; 14.4%], or schizoaffective disorder [29; 9.5%, [Fig 1, “Type of Severe Mental Illness”]. Nearly half of the participants [147; 48.2%] were on antipsychotic medications [Table 1].

**Table 1:** Description of demographic and biopsychosocial factors of participants with severe mental illness attended Bugando Medical Centre from August to October 2024, Mwanza [n = 305].

Characteristics	Frequency, n [%]
**Sex**	
*Male*	179 [58.7]
*Female*	126 [41.3]
	Mean [SD]: 38 [14.2]
**Age group, years**	
*18–24*	53 [17.3]
*25–34*	86 [28.2]
*35–44*	74 [24.3]
*≥ 45*	92 [30.2]
**Marital status**	
*Single*	134 [43.9]
*Married*	130 [42.6]
*Divorced/Separated*	41 [13.4]
**Education level**	
*No formal education*	20 [6.5]
*Primary education*	121 [39.7]
*Secondary education*	85 [27.9]
*College/university education*	79 [25.9]
**Employment status**	
*Unemployed*	231 [75.7]
*Employed*	74 [24.3]
**Health insurance status**	
*No*	222 [72.8]
*Yes*	83 [27.2]
**Distance to the hospital, KM**	
*1–5*	73 [23.9]
*6–10*	31 [10.2]
*> 11*	201 [65.9]
**Duration since diagnosis[years]**	
*1–5*	200 [65.5]
*6–10*	42 [13.8]
*> 11*	63 [20.7]
**Medication group currently in Use**	
*Combination of groups*	139 [45.6]
*Antidepressants*	19 [6.2]
*Antipsychotics*	147 [48.2]

**Note: KM: Kilometers, Combination of groups** *implies that patient was using more than one group of medication including antipsychotics, antidepressants, benzodiazepines, anticonvulsants or mood stabilizers and not the subgroups within these groups.*

### The prevalence of Metabolic Syndrome among patients with severe Mental Illnesses [SMIs]

**Fig 2:**
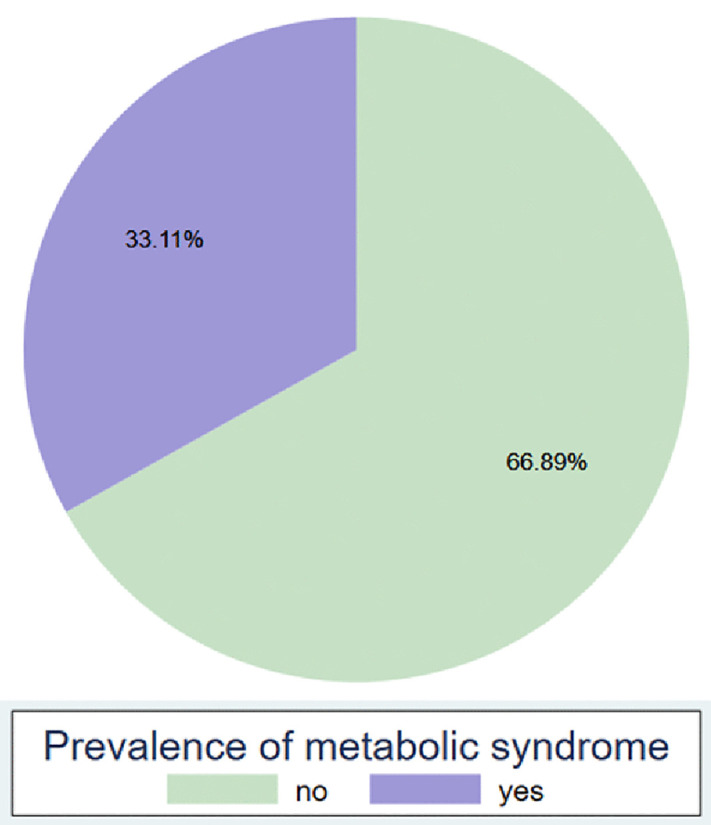
Prevalence of metabolic syndrome.

Of the 305 participants, 101 [33.1%] were found to have metabolic syndrome [Fig 2] [“Prevalence of Metabolic syndrome”].

### The biopsychosocial factors that are associated with Metabolic syndrome among patients with Severe Mental Illnesses [SMIs]

#### Bivariable analysis.

In bivariable analysis showed that several factors were associated with metabolic syndrome (MetS). Females had a higher prevalence of MetS than males (40.5% vs. 27.9%; p = 0.023), and MetS prevalence increased with age, from 9.4% in the 18–24 years group to 46.7% in participants aged ≥45 years (p < 0.001). Marital status, distance from the hospital, type of severe mental illness, and duration since diagnosis were also significantly associated with MetS in bivariable analyses. No significant associations emerged for education level, employment status, health insurance coverage, or medication type. The data were summarized in Table 2 [Table 2].

**Table 2:** Logistic regression models to determine the biopsychosocial factors of patients with severe mental illness attended Bugando Medical Centre from August to October 2024 that are associated with MetS.

Variable	N[%,MetS]	COR [95% CI]	*p-value*	AOR [95% CI]	*p-value*
**Total participants**					
**Sex**				305	
*Male*	50 [27.9]	1		1	
*Female*	51 [40.5]	1.75 [1.08-2.84]	**0.023**	1.46 [0.83-2.57]	0.186
**Age group, years**					
*18–24*	5 [9.4]	1		1	
*25–34*	24 [27.9]	3.72 [1.32-10.46]	0.013	3.21 [1.07-9.69]	0.038
*35–44*	29 [39.2]	6.19 [2.20-17.37]	0.001	4.58 [1.42-14.83]	0.011
*≥ 45*	43 [46.7]	8.42 [3.07-23.08]	**<0.001**	5.15 [1.55-17.16]	**0.008**
**Marital status**					
*Single*	28 [20.9]	1		1	
*Married*	52 [40.0]	2.52 [1.46-4.35]	0.001	1.22 [0.60-2.45]	0.580
*Divorced/Separated*	21 [51.2]	3.98 [1.90-8.34]	**<0.001**	1.42 [0.58-3.48]	0.442
**Education level**					
*None or Informal*	7 [35.0]	1		1	
*Primary education*	42 [34.7]	0.98 [0.36-2.66]	0.980	0.98 [0.32-3.01]	0.968
*Secondary education*	23 [27.1]	0.69 [0.24-1.94]	0.481	0.92 [0.28-2.99]	0.895
*College/university education*	29 [37.7]	1.08 [0.39-3.00]	0.887	1.19 [0.32-4.32]	0.797
**Employment status**					
*Unemployed*	70 [30.3]	1		1	
*Employed*	31 [41.9]	1.66 [0.97-2.85]	0.067	0.77 [0.35-1.69]	0.514
**Health insurance status**					
*No*	70 [31.5]	1		1	
*Yes*	31 [37.4]	1.29 [0.76-2.19]	0.337	0.68 [0.31-1.46]	0.323
**Distance to hospital, KM**					
*1–5*	17 [24.1]	1		1	
*6–10*	7 [22.6]	0.96 [0.35-2.62]	0.938	0.75 [0.25-2.22]	0.601
*> 11*	77 [38.3]	2.05 [1.11-3.77]	**0.022**	1.88 [0.94-3.76]	0.072
**Type of severe mental illness**					
*Bipolar disorder*	8 [21.6]	1		1	
*Major depressive disorder*	18 [40.9]	3.55 [1.34-9.33]	**0.010**	3.15 [0.97-10.22]	0.056
*Schizophrenia*	65 [35.5]	2.82 [1.25-6.38]	**0.013**	1.77 [0.69-4.49]	0.232
*Schizoaffective*	10 [34.5]	2.70 [0.92-7.92]	0.071	1.96 [0.60-6.38]	0.262
**Duration since diagnosis**					
*1–5*	53 [26.5]	1		1	
*6–10*	14 [33.3]	1.39 [0.68-2.83]	0.370	0.86 [0.39-1.92]	0.719
*> 11*	34 [54.0]	3.25 [1.81-5.84]	**<0.001**	1.84 [0.92-3.67]	0.084
**Medications group currently in Use**					
*Combination of groups*	40 [28.8]	1		1	
*Antidepressants*	7 [36.8]	1.44 [0.53-3.93]	0.472	0.78 [0.22-2.79]	0.701
*Antipsychotics*	54 [36.7]	1.44 [0.87-2.36]	0.153	1.30 [0.69-2.45]	0.408

**Note: KM implies Kilometers, Combination of groups** implies that patient was using more than one group of medication that is antipsychotics, antidepressants, benzodiazepine, anticonvulsants or mood stabilizers and not the subgroups within these groups.

#### Multivariable analysis.

After adjusting for confounders in multivariable logistic regression, age remained the only independent predictor of MetS. Participants aged 25–34 years (AOR = 3.21; 95% CI: 1.07–9.69), 35–44 years (AOR = 4.58; 95% CI: 1.42–14.83), and ≥45 years (AOR = 5.15; 95% CI: 1.55–17.16) had significantly higher odds of MetS compared with the 18–24 years age group. Other variables, including sex, marital status, distance to the hospital, type and duration of illness, and current medications, were not independently associated with MetS. The results of the multivariate analysis are summarized in Table 2 below.

## Discussion

This study aimed to determine the prevalence of, and factors associated with, metabolic syndrome among patients with severe mental illness at Bugando Medical Centre, Mwanza. The study also sought to determine if there was an association between the biopsychosocial factors and metabolic syndrome in this patients.

The present study found that 33.1% of participants with severe mental illness [SMI] at Bugando Medical Centre [BMC] had metabolic syndrome [MetS]. This prevalence is notably higher than that of the general African population [12.5%–31.4%], as summarized by Noubiap and colleagues [2022] [18]. This difference may be attributable to the effects of psychotropic medications and other illness-related factors [19]. Additionally, variations in the assessment tools and populations used in systematic reviews of the general population, along with the relatively high burden of comorbidities among patients with SMI, may have contributed to the observed display [18].

The prevalence of metabolic syndrome observed in our study was also higher than that reported among patients with severe mental illness in Kenya [28.6%] [20], Uganda [23.51%] [21] and Ethiopia [24.5% using NCEP-ATP III criteria and 26.9% using IDF criteria] [22]. This difference may be partly explained by the younger study populations in Kenya, Uganda, and Ethiopia, where the mean ages ranged from 30 to 33 years. Younger age is associated with a lower risk of metabolic syndrome, which may account for the relatively lower prevalence reported in these studies.

On the other hand, the prevalence of metabolic syndrome observed in this study was slightly lower than that reported in studies conducted in high income countries. A study conducted in the United States among Hispanic and non-Hispanic patients with schizophrenia found that 63% of all patients with schizophrenia had metabolic syndrome. Specifically, metabolic syndrome was present in 41% of non-Hispanic patients and 74% of Hispanic patients [23]. Similary, a study conducted in Europe, in North Macedonia, reported that 46.0% of participants with SMI had metabolic syndrome [24]. The lower prevalence observed in our study compared with those conducted in high-income countries may be attributable to differences in study design, including smaller sample sizes in the studies by Kato (2004) and Teshome (2020), as well as the restriction of participants to a single diagnostic category of severe mental illness.

Our study found that older age was strongly associated with an increased risk of metabolic syndrome. Several studies have demonstrated that as patients with SMI age, their risk of developing metabolic syndrome increases due to multiple factors. These include longer duration of SMI [25], prolonged periods of active symptoms [19], extended exposure to psychotropics medications and cummulative time spent engaging in unhealthy dietary habbits [26]. Furthermore, studies conducted in Uganda and Kenya similary report a strongly association between increasing age and the development of metabolic syndrome [20,21].

Our study found no significant association between metabolic syndrome and the type of psychotropic medications used by patients with severe mental illness. This may be explained by the fact that the majority of participants were relatively young and had been diagnosed with severe mental illness within the preceding one to five years, suggesting limited cumulative exposure to psychotropic medications. Previous studies have reported conflicting findings regarding the association between psychotropic medication use and metabolic syndrome. For instance, a study conducted in Kenya found no association between olanzapine use and metabolic syndrome [20]. In contrast, studies from high-income countries have demonstrated a significant association between psychotropic medication use and metabolic syndrome [27].

The results of this study did not demonstrate an association between diagnosis of severe mental illness and metabolic syndrome. This may be because the majority of participants were recently diagnosed with schizophrenia, which may have limited the cumulative effects of illness-related and treatment-related risk factors. However, findings from other studies are conflicting. For example a study conducted in Uganda similarily reported no association between severe mental illness and metabolic syndrome [21] whereas studies conducted in high-income countries have demonstrated a significant association [24]. These discrepancies may be explained by differences in study design and areas of focus, as some studies from high-income countries have restricted their analysis to a single category of severe mental illness.

## Recommendations

Based on our findings, we recommend that clinicians raise awareness among patients with severe mental illness and their caregivers about the risk of developing metabolic syndrome and its associated complications. Psychoeducation should be provided on strategies to prevent the development of metabolic syndrome, as well as on the importance of regular screening for early detection. A multidisciplinary approach involving clinicians and allied healthcare professionals is essential for reducing the prevalence of metabolic syndrome among patients with severe mental illness. Furthermore, longitudinal studies are needed to establish causal relationships between specific risk factors and the development of metabolic syndrome and to evaluate the effectiveness of targeted interventions aimed at reducing its prevalence.

## Limitations

There are several limitations that may have affected the findings of our study. Recall bias related to the retrospective, self-reported nature of questionnaire data may have limited the precision with which independent variables were measured. This could have resulted in either over- or under-estimation of the variables assessed. The use of the ICD-10 definition of SMI may also have led to imprecion in estimating prevalence as clinical judgement is required for diagnosis. Furthermore, selection bias may have onfluenced the findings, as only patients who were attending clinic visits were included. The study also relied on self-reported information for certain variables, such as the timing of the last meal, which may be subject to participant reporting errors and could have further limited the accuracy of the results.

## Conclusion

This study revealed that approximately three out of ten participants had metabolic syndrome, indicating a relatively high prevalence among patients with severe mental illness.This findings underscores an increased risk of cardiovascular complications among patients with SMI at BMC. Increasing age, particulary among individuals aged 45 years and older, was associated with higher odds of developing metabolic syndrome compared with younger age groups (18–24 years). These findings highlight the importance of early identification and timely intervention to reduce cardiovascular and other metabolic complications in this vulnerable population.

## Supporting information

S1 Data Minimal data set. (XLSX)

## References

[1] Expert Panel on Detection, Evaluation, and Treatment of High Blood Cholesterol in Adults. Executive Summary of The Third Report of The National Cholesterol Education Program (NCEP) Expert Panel on Detection, Evaluation, And Treatment of High Blood Cholesterol In Adults (Adult Treatment Panel III). JAMA. 2001;285(19):2486–97. doi: 10.1001/jama.285.19.2486 11368702

[2] Grundy SM. Metabolic syndrome pandemic. Arterioscler Thromb Vasc Biol. 2008;28(4):629–36. doi: 10.1161/ATVBAHA.107.151092 18174459

[3] Hennekens CH. Increasing global burden of cardiovascular disease in general populations and patients with schizophrenia. J Clin Psychiatry. 2007;68(4):4. 17539693

[4] Meyer J, Koro CE, L’Italien GJ. The metabolic syndrome and schizophrenia: a review. Int Rev Psychiatry. 2005;17(3):173–80. doi: 10.1080/09540260500071798 16194788

[5] Takeshita J, Masaki K, Ahmed I, Foley DJ, Li YQ, Chen R, et al. Are depressive symptoms a risk factor for mortality in elderly Japanese American men?: the Honolulu-Asia Aging Study. Am J Psychiatry. 2002;159(7):1127–32. doi: 10.1176/appi.ajp.159.7.1127 12091190

[6] Goodwin RD, Davidson JR. Self-reported diabetes and posttraumatic stress disorder among adults in the community. Prev Med. 2005;40(5):570–4. doi: 10.1016/j.ypmed.2004.07.013 15749140

[7] Weber-Hamann B, Hentschel F, Kniest A, Deuschle M, Colla M, Lederbogen F, et al. Hypercortisolemic depression is associated with increased intra-abdominal fat. Psychosom Med. 2002;64(2):274–7. doi: 10.1097/00006842-200203000-00010 11914443

[8] DE Hert M, Correll CU, Bobes J, Cetkovich-Bakmas M, Cohen D, Asai I, et al. Physical illness in patients with severe mental disorders. I. Prevalence, impact of medications and disparities in health care. World Psychiatry. 2011;10(1):52–77. doi: 10.1002/j.2051-5545.2011.tb00014.x 21379357 PMC3048500

[9] Swinson Evans T, Berkman N, Brown C, Gaynes B, Palmieri Weber R. Disparities Within Serious Mental Illness. Technical Brief No. 25. 2016;[25]. Available from: www.ahrq.gov 27336120

[10] Bushe C, Leonard B. Association between atypical antipsychotic agents and type 2 diabetes: review of prospective clinical data. Br J Psychiatry. 2004;184(S47):s87-93. 10.1192/bjp.184.47.s87 15056600

[11] McEvoy JP, Meyer JM, Goff DC, Nasrallah HA, Davis SM, Sullivan L, et al. Prevalence of the metabolic syndrome in patients with schizophrenia: baseline results from the Clinical Antipsychotic Trials of Intervention Effectiveness (CATIE) schizophrenia trial and comparison with national estimates from NHANES III. Schizophr Res. 2005;80(1):19–32. doi: 10.1016/j.schres.2005.07.014 16137860

[12] Bobes J, Arango C, Aranda P, Carmena R, Garcia-Garcia M, Rejas J, et al. Cardiovascular and metabolic risk in outpatients with schizophrenia treated with antipsychotics: results of the CLAMORS Study. Schizophr Res. 2007;90(1–3):162–73. doi: 10.1016/j.schres.2006.09.025 17123783

[13] Lockett H, Cunningham R, Bagnall C, Arcus K. Cardiovascular disease risk assessment and management in people who experience serious mental illness: an evidence review. Heart Lung Circ. 2016;25:S37.

[14] De Hert M, Dekker JM, Wood D, Kahl KG, Holt RIG, Möller H-J. Cardiovascular disease and diabetes in people with severe mental illness position statement from the European Psychiatric Association (EPA), supported by the European Association for the Study of Diabetes (EASD) and the European Society of Cardiology (ESC). Eur Psychiatry. 2009;24(6):412–24. doi: 10.1016/j.eurpsy.2009.01.005 19682863

[15] Mitchell AJ, Vancampfort D, Sweers K, van Winkel R, Yu W, De Hert M. Prevalence of metabolic syndrome and metabolic abnormalities in schizophrenia and related disorders--a systematic review and meta-analysis. Schizophr Bull. 2013;39(2):306–18. doi: 10.1093/schbul/sbr148 22207632 PMC3576174

[16] Saloojee S, Burns JK, Motala AA. Very low rates of screening for metabolic syndrome among patients with severe mental illness in Durban, South Africa. BMC Psychiatry. 2014;14:228. doi: 10.1186/s12888-014-0228-5 25113131 PMC4149236

[17] Hosmer Jr DW, Lemeshow S, Sturdivant RX. Applied logistic regression. John Wiley & Sons. 2013.

[18] Noubiap JJ, Nansseu JR, Lontchi-Yimagou E, Nkeck JR, Nyaga UF, Ngouo AT, et al. Geographic distribution of metabolic syndrome and its components in the general adult population: A meta-analysis of global data from 28 million individuals. Diabetes Res Clin Pract. 2022;188:109924. doi: 10.1016/j.diabres.2022.109924 35584716

[19] Penninx BWJH, Lange SMM. Metabolic syndrome in psychiatric patients: overview, mechanisms, and implications. Dialogues Clin Neurosci. 2018;20(1):63–73. doi: 10.31887/DCNS.2018.20.1/bpenninx 29946213 PMC6016046

[20] Kwobah E, Koen N, Mwangi A, Atwoli L, Stein DJ. Prevalence and correlates of metabolic syndrome and its components in adults with psychotic disorders in Eldoret, Kenya. PLoS One. 2021;16(1):e0245086. doi: 10.1371/journal.pone.0245086 33428652 PMC7799838

[21] Agaba DC, Migisha R, Namayanja R, Katamba G, Lugobe HM, Aheisibwe H, et al. Prevalence and Associated Factors of Metabolic Syndrome among Patients with Severe Mental Illness Attending a Tertiary Hospital in Southwest Uganda. Biomed Res Int. 2019;2019:1096201. doi: 10.1155/2019/1096201 31815121 PMC6877961

[22] Teshome T, Kassa DH, Hirigo AT. Prevalence and associated factors of metabolic syndrome among patients with severe mental illness at Hawassa, southern-Ethiopia. Diabetes, Metab Syndr Obes. 2020;:569–79. 32161483 10.2147/DMSO.S235379 PMC7051251

[23] Kato MM, Currier MB, Gomez CM, Hall L, Gonzalez-Blanco M. Prevalence of Metabolic Syndrome in Hispanic and Non-Hispanic Patients With Schizophrenia. Prim Care Companion J Clin Psychiatry. 2004;6(2):74–7. doi: 10.4088/pcc.v06n0205 15254600 PMC427602

[24] Isjanovski V, Isjanovski I. Преваленција на метаболички синдром кај пациенти со шизофренија во Центарот за ментално здравје “Пролет”, Психијатриска болница – Скопје. Arch Pub Health. 2019;11(1):95–103. doi: 10.3889/aph.2019.2861

[25] Shiferaw WS, Akalu TY, Gedefaw M, Anthony D, Kassie AM, Misganaw Kebede W, et al. Metabolic syndrome among type 2 diabetic patients in Sub-Saharan African countries: A systematic review and meta-analysis. Diabetes Metab Syndr. 2020;14(5):1403–11. doi: 10.1016/j.dsx.2020.07.013 32755843

[26] Mötteli S, Provaznikova B, Vetter S, Jäger M, Seifritz E, Hotzy F. Examining Nutrition Knowledge, Skills, and Eating Behaviours in People with Severe Mental Illness: A Cross-Sectional Comparison among Psychiatric Inpatients, Outpatients, and Healthy Adults. Nutrients. 2023;15(9):2136. doi: 10.3390/nu15092136 37432259 PMC10180535

[27] Ferns G. Cause, consequence or coincidence: the relationship between psychiatric disease and metabolic syndrome. Transl Metab Syndr Res. 2018;1:23–38.

